# Immunogenicity, safety, and reactogenicity of combined reduced-antigen-content diphtheria-tetanus-acellular pertussis vaccine administered as a booster vaccine dose in healthy Russian participants: a phase III, open-label study

**DOI:** 10.1080/21645515.2020.1796423

**Published:** 2020-08-26

**Authors:** Asmik Asatryan, Nadia Meyer, Michael Scherbakov, Victor Romanenko, Irina Osipova, Anna Galustyan, Olga Shamsheva, Tatiana Latysheva, Tatyana Myasnikova, Nathalie Baudson, Monique Dodet, Stebin Xavier, Lauriane Harrington, Anastasia Kuznetsova, Laura Campora, Peter Van den Steen

**Affiliations:** a Hospital Stomamedservice, Gatchina, Russian Federation; b GSK, Wavre, Belgium; c GSK, Moscow, Russia; d Children’s City Hospital No11, Ekaterinburg, Russian Federation; e Ooo “Asko-med-plus”, Barnaul, Russian Federation; f Medical Technologies Ltd, St. Petersburg, Russian Federation; g Federal State Budgetary Educational Institution of Higher Education, St. Petersburg State Pediatric Medical University of the Ministry of Healthcare of the Russian Federation, St. Petersburg, Russian Federation; h Pirogov Russian National Research Medical University, Moscow, Russian Federation; i NRC Institute of Immunology FMBA, Moscow, Russia; j GSK Vaccines, Rixensart, Belgium

**Keywords:** Acellular pertussis, diphtheria, tetanus, booster vaccination, immunogenicity, reactogenicity, safety

## Abstract

As vaccine-induced immunity and protection following natural pertussis infection wane over time, adults and adolescents may develop pertussis and become transmitters to unprotected infants. In Russia, diphtheria and tetanus but not pertussis-containing vaccines are registered for older children, adolescents, or adults. The reduced-antigen-content diphtheria toxoid, tetanus toxoid, and acellular pertussis (dTpa) vaccine (*Boostrix*, GSK) was developed for booster vaccination of children ≥4 years of age, adolescents, and adults. A phase III, open-label, non-randomized study was performed in eight centers in Russia between January and July 2018. The objective of this study was to assess immunogenicity, reactogenicity and safety of a single dose of dTpa vaccine in healthy Russian participants ≥4 years of age (age categories 4–9 years, 10–17 years, 18–64 years, and ≥65 years). At 1 month post-booster vaccination, across all age groups, >99.0% of participants were seroprotected against diphtheria and tetanus and >96.0% of participants were seropositive for anti-pertussis antibodies. For all antibodies across all age groups, antibody GMCs increased from pre- to 1 month post-booster vaccination and booster responses to diphtheria (in 71.5% of participants), tetanus (85.3%), and pertussis antigens (≥85.6%) were observed. One serious adverse event that was not causally related to the study vaccine was reported. No fatal cases were reported throughout the study period. In conclusion, administration of the dTpa vaccine as a booster dose in healthy Russian participants induced a robust immune response to all vaccine antigens and was generally well tolerated across all age groups.

## Introduction

Pertussis or whooping cough is a highly contagious respiratory disease caused by the bacterium *Bordetella pertussis* that may lead to serious or even deadly complications in infants and young children. Pertussis-related deaths in young infants are most commonly associated with secondary bacterial pneumonia.^1,2^ Universal immunization of infants with multiple doses of pediatric diphtheria toxoid, tetanus toxoid, and whole-cell or acellular pertussis (DTP) vaccines has strongly reduced the occurrence of diphtheria, tetanus, and pertussis in infants.^1^ Following primary immunization series, booster DTP doses are recommended to be administered in the second year of life and later at pre-school or early school age.^2,3^ Despite these measures, an estimated 24.1 million pertussis cases and about 160,700 deaths per year in children younger than 5 years of age (YOA) were reported worldwide in 2014.^4^ As vaccine-induced immunity and protection following natural pertussis infection wane over time, adults and adolescents may become a source of infection for unvaccinated or not fully vaccinated infants, the age group with the highest morbidity and mortality.^5–7^

The reduced-antigen-content diphtheria toxoid, tetanus toxoid, and acellular pertussis (dTpa) vaccine (*Boostrix*, GSK) was developed for booster vaccination of children, adolescents, and adults. *Boostrix* was approved for use in 27 countries of the European Union and 54 other countries for booster vaccination in individuals four YOA and older.^8,9^ Boosting with dTpa instead of diphtheria and tetanus toxoids prolongs the immunity against pertussis infection.^10^ This vaccine is used not only for individual protection of vaccinated persons but also for maternal vaccination and to immunize family members and close contacts of newborns in the so-called “cocoon” strategy.^11,12^

In Russia, 10,423 cases of pertussis were reported in 2018 by the World Health Organization Vaccine-Preventable Disease Monitoring System.^13^ Vaccination against pertussis was introduced in Russia in 1959.^14^ According to the national immunization calendar, DTP vaccines are applied for active immunization against diphtheria, tetanus, and pertussis diseases in Russian infants as a 3 + 1 schedule administered at 3, 4.5, 6 months and at 18 months of age, resulting in a coverage rate of 97%.^13,14^ While immunization with tetanus toxoid and reduced diphtheria toxoid vaccine is recommended decennially starting from 6 to 7 YOA,^13^ vaccination against pertussis is not provided for older children, adolescents, and adults. Given the decline in protection following the primary vaccination series, this age category is at increased risk of developing pertussis and may also serve as a potential source of pertussis infection.

In the study presented in this manuscript, we assessed the immunogenicity, reactogenicity, and safety of the dTpa vaccine in healthy Russian participants aged 4 years and older. A summary of the research, clinical relevance, and the impact on the patient population is displayed in **Supplementary Figure 1**.

## Methods

### Study design and participants

**Figure 1.**
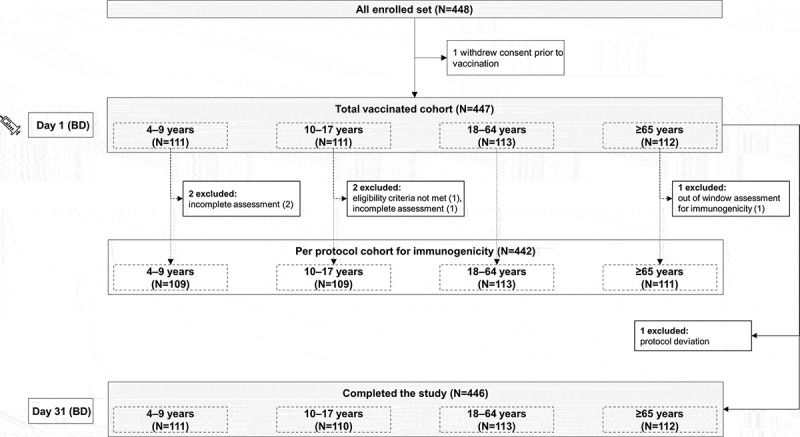
Participants flow diagram

BD, blood draw; N, number of participants. Syringe indicates reduced-antigen-content diphtheria and tetanus toxoid and acellular pertussis (dTpa) vaccine.

This phase III, open-label, non-randomized, single-group study was performed in eight centers in Russia between January and July 2018. Healthy participants, males and females, ≥4 YOA were recruited in the following age categories: 4–9 years (children), 10–17 years (adolescents), 18–64 years (adults) and ≥65 years (elderly population). All enrolled participants received a single dose of dTpa vaccine at Visit 1 (Day 1, Figure 1). The recruitment and age stratification of participants into the study was tracked using GSK Biologicals’ central randomization system on Internet. Participants 4–7 YOA were included if they had received diphtheria, tetanus, and pertussis vaccination (primary series and one booster dose) prior study enrollment but not any further diphtheria-tetanus containing booster vaccine. Participants ≥8 YOA were included if they had received diphtheria, tetanus vaccination (with or without pertussis) more than 5 years prior to the study enrollment. Participants with a history of previous or intercurrent diphtheria, tetanus, or pertussis diseases since birth (4–7 YOA) or within 5 years prior to enrollment (≥8 YOA) were excluded from the study. Detailed exclusion criteria can be found in the **Supplementary material**. Written informed consent was obtained from the participant/participant’s parent(s)/adoptive parent(s) prior to performing any study-specific procedure. Written informed assent was obtained from participants aged 14–<18 years. The study was performed in agreement with the International Conference on Harmonization (ICH) guidelines for good clinical practice, the ICH Harmonized Tripartite Guideline for clinical investigation of medicinal products in the pediatric population, applicable local regulations, and the Declaration of Helsinki. The protocol and the proposed informed consent/assent forms were approved by institutional review board/independent ethics committee. The study is registered at www.clinicaltrial.gov (NCT03311659) and full study protocol (study number: 201532) is available at https://www.gsk-studyregister.com/study/5401.

### Study vaccine

A vaccine dose of 0.5 mL was injected intramuscularly in the deltoid of the non-dominant arm. One dTpa vaccine dose contains ≥2 international units (IU) diphtheria toxoid (D), ≥20 IU tetanus toxoid (T), 8 μg pertussis toxoid (PT), 8 μg filamentous hemagglutinin (FHA), 2.5 μg pertactin (PRN), and 500 μg Aluminum (Al3^+^).

### Objectives

The primary study objective was to assess the immune response to the dTpa vaccine in terms of seroprotection status for antibodies against D and T antigens and in terms of seropositivity status for antibodies against the pertussis antigens (PT, FHA and PRN) 1 month after vaccination. Secondary immunogenicity objectives were to evaluate the booster response and antibody concentrations against D, T, PT, FHA, and PRN antigens 1 month after vaccination. Safety objectives included the assessment of the reactogenicity and safety of dTpa vaccine in terms of solicited (local and general) and unsolicited adverse events (AEs) and serious adverse events (SAEs).

### Immunogenicity assessment

Blood samples of approximately 5 ml (whole blood) were collected from all participants pre-vaccination (during Visit 1 at Day 1) and one-month post-vaccination. Antibodies against D, T, PT, FHA, and PRN were assessed by validated enzyme-linked immunosorbent assay (ELISA). Assay cutoffs were 0.057 IU/ml for anti-D, 0.043 IU/ml for anti-T, 2.693 IU/ml for anti-PT, 2.046 IU/ml for anti-FHA, and 2.187 IU/ml for anti-PRN. Seroprotection against diphtheria and tetanus was defined as antibody concentrations ≥0.1 IU/ml.^15,16^ If anti-D ELISA antibody concentrations were <0.1 IU/ml, the Vero-cell neutralization assay was performed for pre- and post-vaccination serum samples (assay cutoff 0.004 IU/ml). Antibody concentrations ≥0.01 IU/ml were considered as protective.^17^ Both the ELISA test (antibody concentrations ≥0.1 IU/ml) and Vero-cell test (antibody concentration ≥0.01 IU/ml) were used to define the seroprotection status for the primary endpoint.

No serological correlate of protection is defined for the immune response to pertussis antigens. Participants with anti-PT, anti-FHA, and anti-PRN antibody concentrations above the assay cutoffs were considered seropositive.

### Safety assessment

Solicited local and general AEs occurring within 4 days (Day1–4) post-vaccination and unsolicited AEs occurring during the 31 days (Day1–31) post-vaccination period were recorded on diary cards by the participants/participants’ parent(s)/adoptive parent(s). If a large swelling site reaction was observed during the 4 days follow-up period after vaccination, the participant or participant’s parent(s)/adoptive parent(s) had to contact the study personnel promptly and visit the investigator’s office at the earliest opportunity. Detailed information describing the AE was recorded by the investigator or delegate on a specific large injection site reaction sheet in the electronic case report form. Medically attended AEs, AEs leading to study withdrawal, and SAEs were collected throughout the study. AEs were graded by severity (from mild to severe) and their relationship to study vaccination was assessed by the investigators. Criteria for Grade 3 AE are listed in the footnotes of figures and tables presenting Grade 3 AE results. Large swelling was defined as a swelling with a diameter of >50 mm for participants <6 YOA and diameter of >100 mm, a noticeable diffuse swelling, or a noticeable increase in limb circumference for participants ≥6 YOA. Although pregnant women or women planning to become pregnant during the study period were excluded (see **Supplementary material**), any pregnancy occurring after vaccination had to be recorded on an electronic pregnancy report. Pregnant women could continue the study at the discretion of the investigator.

### Statistical analysis

Assuming a drop-out rate of 10% to compensate for participant attrition due to early study withdrawal, a total of 448 participants (112 in each age group) were to be enrolled in the study in order to obtain the desired number of evaluable participants for analysis. The primary immunogenicity analysis was based on the per protocol cohort for immunogenicity. If the percentage of vaccinated participants with serological results excluded from the per protocol cohort for analysis of immunogenicity was ≥5%, a second analysis based on the total vaccinated cohort was performed including all participants vaccinated for whom data concerning at least one immunogenicity endpoint were available. The safety analyses were performed on the total vaccinated cohort, including all participants with the study vaccine administration documented.

We observed participants having post-vaccination results below pre-vaccination results for at least one of the five assays (D, T, PT, FHA, PRN), mainly for one site (see details in the result section). Therefore, this site was regarded an “outlier” site. Sensitivity analysis of the immunogenicity data was performed on the per protocol cohort for immunogenicity excluding participants from the outlier site and results were compared with the sensitivity analysis conducted on all participants included in the per protocol cohort for immunogenicity.

Demographic characteristics were summarized using descriptive statistics. For all participants and each antigen, seropositivity/seroprotection rates at pre-vaccination and 1 month post-vaccination were calculated with exact 95% confidence intervals (CIs). Booster response rates 1 month post-vaccination were calculated with exact 95% CIs for each antigen. In addition, the above analyses were also performed based on age stratification.

A booster response to D and T antigens was defined as antibody concentrations ≥0.4 IU/ml 1 month after vaccination for participants with pre-vaccination antibody concentration <0.1 IU/ml (i.e., below the seroprotection cutoff) and as an increase in antibody concentrations of at least four times the pre-vaccination concentration 1 month after vaccination for participants with pre-vaccination antibody concentration ≥0.1 IU/ml (i.e., equal to or above the seroprotection cutoff). A booster response to PT, FHA, and PRN antigens was defined as post-vaccination antibody concentration ≥4 times the assay cutoff for participants with pre-vaccination antibody concentration below the cutoff, post-vaccination antibody concentration ≥4 times the pre-vaccination antibody concentration for participants with pre-vaccination antibody concentration between the assay cutoff and below 4 times the assay cutoff, and post-vaccination antibody concentration ≥2 times the pre-vaccination concentration for participants with pre-vaccination antibody concentration ≥4 times the assay cutoff. The geometric mean concentration (GMC) calculations were performed by taking the anti-log of the mean of the log_10_ concentration transformations. Antibody concentrations below the assay cutoff were given an arbitrary value of half the cutoff for the purpose of GMC calculation.

## Results

### Demographic characteristics

Of the 448 participants enrolled in the study, 447 (111 [group 4–9 years], 111 [group 10–17 years], 113 [group 18–64 years] and 112 [group ≥65 years]) were included in the total vaccinated cohort and 442 (109 [group 4–9 years], 109 [group 10–17 years], 113 [group 18–64 years] and 111 [group ≥65 years]) in the per protocol cohort. Overall, 446 participants completed the study. One participant withdrew prior to vaccination; five participants were excluded from the per protocol cohort for immunogenicity. Reasons for exclusion are listed in Figure 1.

The median age of participants was 19 years (minimum 4; maximum 96) and number of participants per age group was similar. Overall, gender was balanced, although more females (53.9%) were enrolled in the study (Table 1).

**Table 1.** Demographic and baseline characteristics (total vaccinated cohort)

	dTpa Group N = 447
Age, years at vaccination	
Mean±SD (years)	32.7 ± 27.3
Median (minimum; maximum)	19 (4;96)
Age group, n (%)	
4–9 years	111 (24.8)
4–5 years	18 (4.0)
6–9 years	93 (20.8)
10–17 years	111 (24.8)
18–64 years	113 (25.3)
≥65 years	112 (25.1)
Gender, n (%)	
Male	206 (46.1)
Female	241 (53.9)
Geographic ancestry	
White-Caucasian/European Heritage, n (%)	447 (100)

N, number of participants with available data; n (%), number (percentage) of participants in each category; SD, standard deviation; dTpa, diphtheria-tetanus-acellular pertussis.

### Immunogenicity

At 1 month post-booster vaccination, across all age groups, >99.0% of participants were seroprotected against D and T (antibody concentrations ≥0.1 IU/mL) and >97% of participants were seropositive for anti-pertussis antibodies (antibody concentrations ≥assay cutoff). In the 4–9 years, 10–17 years, and 18–64 years age groups all participants were seroprotected against D and T, while in ≥65 years age group, >99.0% and >98.0% of participants were seroprotected against D and T, respectively (Table 2). The participant in the ≥65 years age group who did not reach seroprotection against D as measured by the ELISA assay, was shown to have protective levels of anti-D neutralizing antibodies using the more sensitive Vero-cell neutralization assay. Therefore, all study participants were considered seroprotected at 1 month post-vaccination.

**Table 2.** Seroprotection rate for diphtheria and tetanus antibodies and seropositivity rates for pertussis antibodies at pre-vaccination and at 1 month after dTpa vaccination by age group (per protocol cohort for immunogenicity)

		4–9 years			10–17 years			18–64 years			≥65 years		
Antibody (cutoff)	Timing	N	n	Seroprotection/ seropositivity % (95%CI)	N	n	Seroprotection/ seropositivity % (95%CI)	N	n	Seroprotection/ seropositivity % (95%CI)	N	n	Seroprotection/ seropositivity % (95%CI)
Anti-D antibody (≥ 0.1 IU/mL)	Pre	107	101	94.4 (88.2; 97.9)	106	95	89.6 (82.2; 94.7)	112	110	98.2 (93.7; 99.8)	108	94	87.0 (79.2; 92.7)
	Post	108	108	100.0 (96.6; 100.0)	109	109	100.0 (96.7; 100.0)	112	112	100.0 (96.8; 100.0)	109	108	99.1^a^ (95.0; 100.0)
Anti-T antibody (≥ 0.1 IU/mL)	Pre	109	99	90.8 (83.8; 95.5)	109	106	97.2 (92.2; 99.4)	112	106	94.6 (88.7; 98.0)	111	87	78.4 (69.6; 85.6)
	Post	109	109	100.0 (96.7; 100.0)	109	109	100.0 (96.7; 100.0)	112	112	100.0 (96.8; 100.0)	111	109	98.2 (93.6; 99.8)
Anti-FHA antibody (≥ 2.046 IU/mL)	Pre	109	105	96.3 (90.9; 99.0)	109	108	99.1 (95.0; 100.0)	113	111	98.2 (93.8; 99.8)	111	110	99.1 (95.1; 100.0)
	Post	109	109	100.0 (96.7; 100.0)	109	109	100.0 (96.7; 100.0)	113	113	100.0 (96.8; 100.0)	111	111	100.0 (96.7; 100.0)
Anti-PRN antibody (≥ 2.187 IU/mL)	Pre	108	81	75.0 (65.7; 82.8)	109	91	83.5 (75.2; 89.9)	112	107	95.5 (89.9; 98.5)	111	92	82.9 (74.6; 89.4)
	Post	109	107	98.2 (93.5; 99.8)	106	105	99.1 (94.9; 100.0)	112	111	99.1 (95.1; 100.0)	109	108	99.1 (95.0; 100.0)
Anti-PT antibody (≥ 2.693 IU/mL)	Pre	109	64	58.7 (48.9; 68.1)	108	55	50.9 (41.1; 60.7)	113	76	67.3 (57.8; 75.8)	110	85	77.3 (68.3; 84.7)
	Post	109	106	97.2 (92.2; 99.4)	107	106	99.1 (94.9; 100.0)	113	111	98.2 (93.8; 99.8)	111	107	96.4 (91.0; 99.0)

CI, confidence interval; D, diphtheria; T, tetanus; dTpa, diphtheria-tetanus-acellular pertussis vaccine; FHA, filamentous hemagglutinin; IU, international unit; N, number of infants with pre- and post-vaccination results available; n (%), number (percentage) of participants who were seroprotected/seropositive (antibody concentration equal to or above the cutoff); pre, Pre-booster blood sampling time point, post, 1 month post-booster blood sampling time point; PRN, pertactin; PT, pertussis toxoid.

^a^Vero-cell assay was used in addition to the enzyme-linked immunosorbent assay and one patient was found to be seroprotective.

Across all age groups, all participants were seropositive for anti-FHA antibodies at 1 month post-booster vaccination; >98.0% of children in 4–9 years age group and >99.0% of participants in 10–17 years, 18–64 years, and ≥65 years age groups were seropositive for anti-PRN antibodies. For anti-PT antibodies, >97.0%, >99.0%, >98.0%, and >96.0% were seropositive in 4–9 years, 10–17 years, 18–64 years, and ≥65 years age groups, respectively (Table 2).

**Figure 2.**
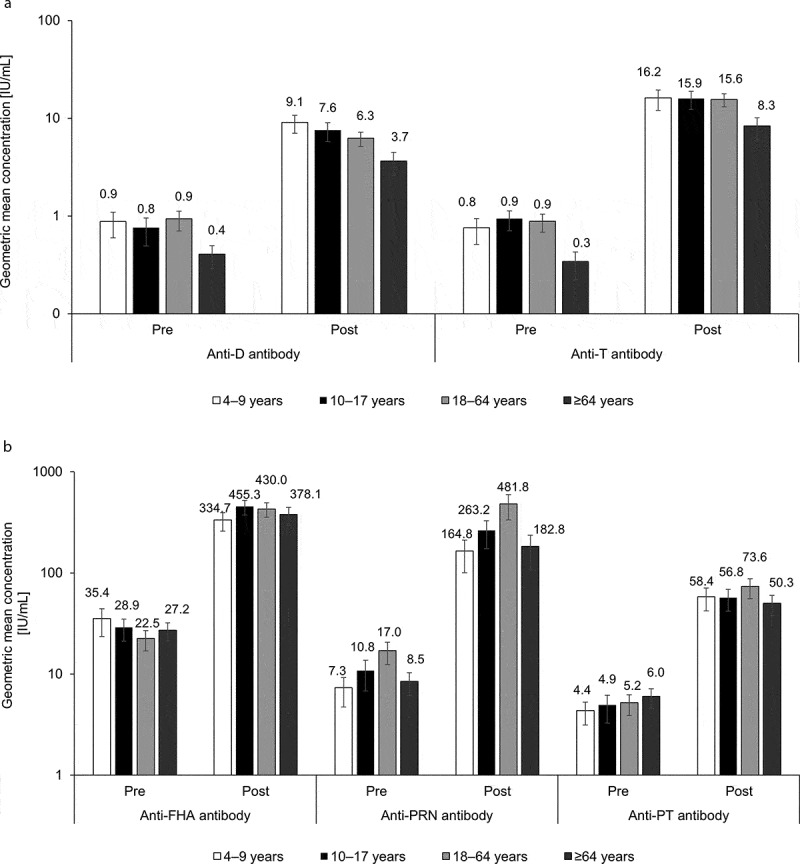
Geometric mean concentration at pre-vaccination and 1 month post-vaccination by age group for anti-D and anti-T antibodies (A) and anti-pertussis antibodies (B) (per protocol cohort for analysis of immunogenicity)

For all antibodies across all age groups, antibody GMCs increased from pre-vaccination to 1 month post-booster vaccination (Figure 2). One month after vaccination, the GMC fold increases from pre- to post-vaccination were 8.80, 19.89, 14.14, 24.48, and 11.56 for anti-D, anti-T, anti-FHA, anti-PRN, and anti-PT antibodies, respectively. A lower pre- and post-vaccination concentration was observed for anti-D and anti-T in the ≥65 years age group.

D, diphtheria; dTpa, diphtheria-tetanus-acellular pertussis vaccine; FHA, filamentous hemagglutinin; IU, international unit; PRN, pertactin; PT, pertussis toxoid, T, tetanus. Error bars represent 95% confidence intervals. The y-axis is log-scaled.

At 1 month post-booster vaccination, a booster response to D, T, PT, FHA, and PRN antigens was observed in 71.5%, 85.3%, 85.6%, 92.8%, and 91.2% of participants, respectively (Table 3).

**Table 3.** Booster response to diphtheria, tetanus, and pertussis antigens 1 month after dTpa vaccination (per protocol cohort for immunogenicity)

	dTpa Group	
Antibody	N	Booster response^a^, % (95% CI)
Anti-D antibody	431	71.5 (66.9; 75.7)
Anti-T antibody	441	85.3 (81.6; 88.4)
Anti-FHA antibody	442	92.8 (89.9; 95.0)
Anti-PRN antibody	434	91.2 (88.2; 93.7)
Anti-PT antibody	438	85.6 (82.0; 88.8)

%, percentage of infants who mounted a booster response; CI, confidence interval; D, diphtheria; T, tetanus; dTpa, diphtheria-tetanus-acellular pertussis; FHA, filamentous hemagglutinin; N, number of participants with both pre- and post-vaccination results available; PRN, pertactin; PT, pertussis toxoid.

^a^Booster response to D and T antigens was defined as: For initially seronegative participants, post-vaccination antibody concentration ≥0.4 IU/mL; for initially seropositive participants, post-vaccination antibody concentration with an increase of at least four times the pre-vaccination antibody concentration. Booster response to pertussis antigens is defined as: For initially seronegative participants, post-vaccination antibody concentration ≥four times the assay cutoff; for initially seropositive participants with antibody concentration <four times assay cutoff, post-vaccination antibody concentration ≥four times the pre-vaccination antibody concentration; and for initially seropositive participants with antibody concentration ≥four times the assay cutoff, post-vaccination antibody concentration ≥two times the pre-vaccination antibody concentration. Assay cutoff was 2.046 IU/mL, 2.187 IU/mL and 2.693 IU/mL for anti-FHA, anti-PRN and anti-PT, respectively.

#### Sensitivity analysis

Laboratory results revealed 43 participants with post-vaccination antibody titers below those pre-vaccination for at least one of the 5 assays performed for antigens PT, FHA, PRN, D (ELISA), and T. An analysis of the study processes (including on-site and laboratory processes) could not identify a root cause. The majority (69.0%) of these observations were reported in the outlier site, impacting 26 of the 43 participants (60.0%) with pre- and post-vaccination results at this site. Sensitivity analysis did not show any effect of the outlier site on the immunogenicity results for the primary and secondary outcomes of the study. Immunogenicity results (mean antibody concentrations and proportion of seroprotected/seropositive participants) with or without including participants from the outlier site were similar (**Supplementary Figure 2**).

### Safety

**Figure 3.**
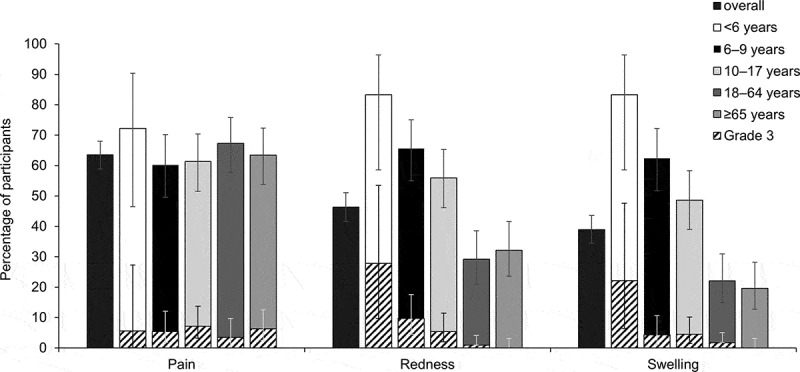
Percentage of participants with solicited local adverse events or unsolicited reported during the 4-day (Days 1–4) period after dTpa vaccination (total vaccinated cohort)

The most frequently reported solicited local AEs were redness and swelling (83.3%) in participants <6 YOA, redness (65.6%) in participants 6–9 YOA, and pain in participants aged ≥10 years (61.3% in 10–17 years age group; 67.3% in 18–64 years age group; 63.4% in ≥65 years age group) (Figure 3 and **Supplementary Table 1**). A large injection site reaction was reported by one (0.2%,1/447) participant in the 18–64 years age group. The large and diffused swelling reached a maximum diameter of 105 mm, did not involve the adjacent joint, was present on day three and four, and resolved thereafter.

dTpa, diphtheria-tetanus-acellular pertussis vaccine. For children <6 years of age, grade 3 pain was defined as crying when the limb was moved or the limb being spontaneously painful and grade 3 redness and swelling as maximum intensity of local redness/swelling with a diameter >20 mm; for children and adults ≥6 years of age grade 3 pain was defined as significant pain at rest, preventing normal everyday activities and redness and swelling as maximum intensity of local redness/swelling with a diameter >50 mm. Error bars represent 95% confidence intervals.

The most frequently reported solicited general AE was irritability/fussiness (27.8%) in participants <6 YOA, and fatigue (29.4%) and headache (24.9%) in all age groups of participants ≥6 YOA (Table 4). Across age groups, 10 (2.2%) participants reported at least one case of fever: 3 (3.2%) participants in the 6–9 years age group, 5 (4.5%) in 10–17 years age group, and one (0.9%) participants in each of the 18–64 years and ≥65 years age groups. All cases were considered by the investigators to be related to the study vaccine. No fever was recorded in children 4–5 YOA.

**Table 4.** Percentage of subjects with solicited general adverse events during the 4-day (Days 1–4) period after dTpa vaccination by age group (total vaccinated cohort)

dTpa Group subset with age <6 years, (N = 18)			dTpa Group subset with age ≥6 years, (N = 429)		
Adverse event	Type	% (95% CI)	Adverse event	Type	% (95% CI)
Drowsiness	All	5.6 (0.1; 27.3)	Fatigue	All	29.4 (25.1; 33.9)
	Related	5.6 (0.1; 27.3)		Related	21.4 (17.7; 25.6)
	Grade 3 Related	0.0 (0.0; 18.5)		Grade 3 Related	1.4 (0.5; 3.0)
Irritability/Fussiness	All	27.8 (9.7; 53.5)	Gastrointestinal symptoms	All	7.9 (5.6; 10.9)
	Related	22.2 (6.4; 47.6)		Related	3.5 (2.0; 5.7)
	Grade 3 Related	0.0 (0.0; 18.5)		Grade 3 Related	0.2 (0.0; 1.3)
Loss of appetite	All	16.7 (3.6; 41.4)	Headache	All	24.9 (20.9; 29.3)
	Related	16.7 (3.6; 41.4)		Related	18.9 (15.3; 22.9)
	Grade 3 Related	0.0 (0.0; 18.5)		Grade 3 Related	2.1 (1.0; 3.9)
Fever	All	0.0 (0.0; 0.0)	Fever	All	2.2 (1.1; 4.1)
	Related	0.0 (0.0; 0.0)		Related	2.2 (1.1; 4.1)
	Grade 3 Related	0.0 (0.0; 0.0)		Grade 3 Related	0.0 (0.0; 0.8)

N; number of participants with the documented dose; %, percentage of participants reporting the specified adverse event at least once; 95% CI; exact 95% confidence interval; dTpa, diphtheria-tetanus-acellular pertussis; related; adverse events, which is assessed by the investigator as related to vaccination; all, all reports of the specified adverse event irrespective of intensity grade and relationship to vaccination.

Fever was defined as temperature ≥38.0°C. The preferred location for measuring temperature in this study was the axilla.

Grade 3 irritability was defined as crying that could not be comforted or irritability preventing normal activity; grade 3 drowsiness as drowsiness preventing normal activity; grade 3 loss of appetite as not eating at all; grade 3 fever was defined as temperature ≥40.0°C; fatigue, gastrointestinal symptoms and headache were considered grade 3 if they prevented normal activity.

During the 31-day follow-up period, unsolicited AEs were reported by 11.6% (52/447) of participants. Grade 3 unsolicited AEs were reported by 1.6% (7/447) of participants. The most frequently reported Grade 3 AE was headache (0.7%, 3/447). Causally related unsolicited AEs, as per investigator’s judgment, were reported by 16 (3.6%) participants; injection site pruritus and cough were the most common ones reported each by 2 (0.4%) participants. AEs that required medical attention were reported by 16 (3.6%) participants during the 31-day follow-up period out of which tracheitis (0.7% 3/447), rhinitis (0.4%, 2/447) and nasopharyngitis (0.4%, 2/447) were most frequently reported. One (0.2%) participant in 18–64 years age groups reported a grade 3 unsolicited AE (somnolence) that was considered by the investigator to be causally related to vaccination (Table 5). One SAE (bacterial pneumonia) was reported for one 16-year-old participant. It was considered as not related to the vaccination by the investigator and the participant fully recovered. No fatal cases were reported throughout the study period. Detailed data per age group are listed in Table 5.

**Table 5.** Percentage of participants with reported unsolicited adverse events and serious adverse events during the 31-day (Days 1–31) period after dTpa vaccination overall and by age group (total vaccinated cohorts)

	Overall N = 447		4–9 years N = 111		10–17 years N = 111		18–64 years N = 113		≥65 years N = 112	
	n	% (95% CI)	n	% (95% CI)	n	% (95% CI)	n	% (95% CI)	n	% (95% CI)
Any unsolicited AEs	52	11.6 (8.8–15.0)	17	15.3 (9.2–23.4)	10	9.0 (4.4–15.9)	19	16.8 (10.4–25.0)	6	5.4 (2.0–11.3)
Grade 3	7	1.6 (0.6–3.2)	0	0.0 (0.0–0.0)	1	0.9 (0.0–4.9)	5	4.4 (1.5–10.0)	1	0.9 (0.0–4.9)
Related to vaccination	16	3.6 (2.1–5.7)	4	3.6 (1.0–9.0)	4	3.6 (1.0–9.0)	6	5.3 (2.0–11.2)	2	1.8 (0.2–6.3)
Grade 3 related to vaccination	1	0.2 (0.0–1.2)	0	0.0 (0.0–3.3)	0	0.0 (0.0–3.3)	1	0.9 (0.0–4.8)	0	0.0 (0.0–3.2)
Medically attended AEs	16	3.6 (2.1–5.7)	8	7.2 (3.2–13.7)	4	3.6 (1.0–9.0)	4	3.5 (1.0–8.8)	0	0.0 (0.0–3.2)
SAEs up to study end, n (%)	1 (0.2)		0 (0)		1 (0.9)		0 (0)		0 (0)	

N, number of participants; n (%), number (percentage) of participants reporting at least one AE; AE, adverse event; CI, confidence interval; dTpa, diphtheria-tetanus-acellular pertussis vaccine; SAE, serious AE.

## Discussion

The dTpa vaccine induced robust immune responses to all vaccine antigens across all age groups. The exclusion of participants from the outlier site had no impact on the overall immunogenicity conclusions. The data for Russian children participating in this study are in line with published data for children 4–6^18^ and 6–8^19^ YOA demonstrating that booster vaccination with dTpa is highly immunogenic. Similarly, adolescents (10–17 years) showed seroprotection/seropositivity rates for anti-D, anti-T, and anti-pertussis antibodies comparable to those observed in previous studies for adolescents.^20,21^ For adults, seroprotection/seropositivity rates for anti-D, anti-T, and anti-pertussis antibodies were high and comparable to those observed in other adult studies.^22–24^

Protective levels against D and T slightly decreased by age, but remained high in the ≥65 years age group (87.0% and 78.4%, respectively). A single dose of dTpa vaccine could effectively boost the immune response in all age groups with a GMC fold increase of at least 8.80. In line with the lower general immunity in older adults,^25^ post-vaccination GMC levels against D and T tended to be slightly lower in participants ≥65 years of age than for younger participants. Nevertheless, antibody levels were indicative of seroprotection and the differences observed were not considered to be of clinical relevance.

Vaccination against pertussis was introduced in Russia in 1959 and is scheduled as 3 priming doses at 3, 4.5, and 6 months and a booster dose at 18 months^14^ and consisted of whole-cell pertussis vaccine as acellular pertussis components were not licensed in Russia before 2007. While pertussis vaccine coverage rates dropped in the 1980s and 1990s resulting in an epidemic pertussis peak in 1994, they were recovered reaching 95% in 2001 and remained above this percentage since then.^26^ However, despite high vaccine coverage in infants, pertussis persisted in certain areas of Russia, underlining the importance of adequate vaccination, in particular in older age groups.^26,27^ In the current study, most participants had detectable anti-pertussis antibodies prior to dTpa vaccination, perhaps from prior vaccination or natural exposure. Pre-vaccination titers did not suggest any age effect, and a single dTpa dose could effectively boost the immune response across all age groups. Similar findings were reported in previous follow-up studies conducted in adolescents^28^ and adults^23,29^ who received a new booster dTpa dose 9 to 10 years later.

The vaccine was generally well tolerated, and no safety concerns were raised in this study. This is in line with the published safety profile of the vaccine.^23,24,28,30^ In the current study, redness and swelling were the most common solicited local AEs reported in children <6 YOA, followed by pain. In children 6–9 YOA, redness was most commonly reported followed by swelling and pain. In other clinical trials, pain was the most frequent solicited AE amongst children 4–6^31^ and 6–8^19^ YOA. Generally, less redness and swelling were reported for older participants (adults and the elderly) in the current study, while injection site pain remained stable across age groups. In line with these observations, pain was the most frequently reported solicited local AE in recently published results for adolescents^20,21^ and adults.^22^ Large injection site swelling reactions have been described for other D and T vaccines^32-34^ and in the label of the GSK’s dTpa vaccine.^35^ One adult reported a large swelling in the current study, which resolved within 2–3 days without sequelae. This is consistent with literature data, as reported in the study published by Pichichero *et al*.^20^ Fatigue and headache were the most common solicited general AEs in participants of all age groups >6 years. In participants ≥65 years of age, the events of fatigue and headache were considered unrelated to vaccination in about 50% of these events. Cases associated with vaccination were comparable or even less frequent in the elderly than in adults 18–64 years of age. Fever did not occur in infants <6 YOA and was present in less than 5% of participants across all groups >6 years of age. Variability in reporting rates of solicited AEs is expected across age as maturity and aging of the immune system and of its physiological functions have been shown to influence susceptibility to adverse reactions to vaccination.^36^

The study was limited by its single-country, open-labeled, non-randomized design. Strengths of the study are the successful inclusion of different age groups and the successful enrollment of a large number of patients from eight different sites belonging to different federal districts of the Russian Federation. The study further benefitted from the use of one standardized study protocol among the sites, the laboratory testing conducted in one central laboratory, the use of diary cards to collect solicited symptoms in a systematic manner, and the use of validated laboratory tests that were calibrated against internationally recognized reference assays.

## Conclusions

The dTpa vaccine *Boostrix* administered as a booster vaccine dose in healthy Russian participants aged 4 years and older induced a robust immune response to all vaccine antigens and was generally well tolerated in all age groups.

## Supplementary Material

Supplemental Material

## Abbreviations

DTP: diphtheria, tetanus toxoids and pertussis
dTpa: reduced-antigen-content diphtheria, tetanus toxoid and acellular pertussis vaccine
DTPa: diphtheria, tetanus toxoids acellular pertussis
D: diphtheria toxoid
T: tetanus toxoid
ELISA: enzyme-linked immunosorbent assay
FHA: filamentous hemagglutinin
GMC: geometric mean concentration
GMT: geometric mean titer
ICH: International Conference on Harmonization
IU: international unit
YOA: years of age
CI: confidence interval
PRN: pertactin
PT: pertussis toxoid
(S)AE: (serious) adverse event.

## Trademark statement

*Boostrix* is a trademark owned by or licensed to the GSK group of companies.

## Data sharing statement

Protocol summary is available at http://www.gsk-clinicalstudyregister.com (study number 201532). For interventional studies that evaluate our medicines, anonymized patient-level data and study documents will be made available to independent researchers, subject to review by an independent panel, at www.clinicalstudydatarequest.com within 6 months of publication. To protect the privacy of patients and individuals involved in our studies, GSK does not publicly disclose patient-level data.
