# Supplementary material for: Immunogenicity, safety, and reactogenicity of combined reduced-antigen-content diphtheria-tetanus-acellular pertussis vaccine administered as a booster vaccine dose in healthy Russian participants: a phase III, open-label study
Source: Hum Vaccin Immunother. 2020 Aug 26;17(3):723–30. doi: 10.1080/21645515.2020.1796423 (PMC7993191; doi:10.1080/21645515.2020.1796423)
Supplement: Supplemental Material [file KHVI_A_1796423_SM2366.docx]

**Supplementary material**

**Immunogenicity, Safety and Reactogenicity of Combined Reduced-Antigen-Content Diphtheria-Tetanus-Acellular Pertussis Vaccine Administered as a Booster Vaccine Dose in Healthy Russian Participants: A Phase III, Open-Label Study**

Asmik Asatryan^a^, Nadia Meyer^b^, Michael Sherbakov^c^, Victor Romanenko^d^, Irina Osipova^e^, Anna Galustyan^f^, Olga Shamsheva^g^, Tatiana Latysheva^h^, Tatyana Myasnikova^h^, Nathalie Baudson^i^, Monique Dodet^b^, Stebin Xavier^b^, Lauriane Harrington^b^, Anastasia Kuznetsova^b^, Laura Campora^b^, Peter Van den Steen^b^*

**Exclusion criteria**

If any of the following criteria applied, the participant was excluded from the study

- Child in care
- Use of any investigational or non-registered product (drug or vaccine) other than the study vaccine during the period starting 30 days before the dose of study vaccine, or planned use during the study period.
- History of previous or intercurrent diphtheria, tetanus or pertussis diseases since birth in participants four to seven years of age.
- History of previous or intercurrent diphtheria, tetanus or pertussis diseases within five years prior to enrolment in participants aged eight years and above.
- Chronic administration (defined as more than 14 days in total) of immunosuppressants or other immune-modifying drugs within six months prior to the vaccine dose. For corticosteroids, this will mean prednisone ≥20 mg/day (for adult participants, ≥18 years of age) or ≥0.5 mg/kg/day (for paediatric participants, aged 4–17 years), or equivalent. Inhaled and topical steroids are allowed.
- Administration of long-acting immune-modifying drugs at any time during the study period (e.g. infliximab).
- Planned administration/administration of a vaccine not foreseen by the study protocol within the period starting 30 days before and ending 30 days after the dose of vaccine with the exception of inactivated influenza vaccine which can be given at any time during the study conduct as per the Summary of Product Characteristics (SmPC) and according to the local governmental recommendations.
- Concurrently participating in another clinical study, at any time during the study period, in which the participant has been or will be exposed to an investigational or a non-investigational vaccine/product (pharmaceutical product or device).
- Any confirmed or suspected immunosuppressive or immunodeficient condition, based on medical history and physical examination (no laboratory testing required).
- Family history of congenital or hereditary immunodeficiency.
- History of any reaction or hypersensitivity likely to be exacerbated by any component of the vaccine.
- Hypersensitivity to latex.
- History of encephalopathy (e.g. coma, decreased level of consciousness, prolonged seizures) after administration of a previous dose of pertussis vaccine that could not be attributed to another identifiable cause, progressive neurologic disorder, uncontrolled epilepsy or progressive encephalopathy: pertussis vaccine should not be administered to individuals with these conditions until a treatment regimen has been established and the condition has stabilized.
- Acute disease and/or fever at the time of enrolment.
- Fever is defined as temperature ≥38.0°C. The preferred location for measuring temperature in this study will be the axilla.
- Participants with a minor illness (such as mild diarrhoea, mild upper respiratory infection) without fever may, be enrolled at the discretion of the investigator.
- Acute or chronic, clinically significant uncontrolled pulmonary, cardiovascular, hepatic or renal functional abnormality, as determined by physical examination and/or laboratory screening tests.
- Administration of immunoglobulins and/or any blood products within the three months preceding the dose of study vaccine or planned administration during the study period.
- Pregnant or lactating female.
- Female planning to become pregnant or planning to discontinue contraceptive precautions during the study conduct.
- Any medical condition that, in the opinion of the investigator, might interfere with the evaluations required by the study.
- Any medical condition that in the judgment of the investigator would make intramuscular injection unsafe.

# Supplementary table 1. Percentage of participants with solicited local adverse events during the 4-day (Days 1-4) period after dTpa administration by age group (total vaccinated cohort)

|  |  | **Overall** | | **<6 years** | |  | **6–9 years** | |  | **10–17 years** | |  | **18–64 years** | | **≥65 years** | |
| --- | --- | --- | --- | --- | --- | --- | --- | --- | --- | --- | --- | --- | --- | --- | --- | --- |
| **Adverse event** | **Timing** | **N** | **% (95%CI)** | **N** | **% (95%CI)** |  | **N** | **% (95%CI)** |  | **N** | **% (95%CI)** |  | **N** | **% (95%CI)** | **N** | **% (95%CI)** |
| Pain | All | 447 | 63.5 (58.9; 68) | 18 | 72.2 (46.5; 90.3) |  | 93 | 60.2 (49.5; 70.2) |  | 111 | 61.3 (51.5; 70.4) |  | 113 | 67.3 (57.8; 75.8) | 112 | 63.4 (53.8; 72.3) |
|  | Grade 3 |  |  | 18 | 5.6 (0.1; 27.3) |  | 93 | 5.4 (1.8; 12.1) |  | 111 | 7.2 (3.2; 13.7) |  | 113 | 3.5 (1; 8.8) | 112 | 6.3 (2.5; 12.5) |
| Redness | All | 447 | 46.3 (41.6; 51.1) | 18 | 83.3 (58.6; 96.4) |  | 93 | 65.6 (55; 75.1) |  | 111 | 55.9 (46.1; 65.3) |  | 113 | 29.2 (21; 38.5) | 112 | 32.1 (23.6; 41.6) |
|  | Grade 3 |  |  | 18 | 27.8 (9.7; 53.5) |  | 93 | 9.7 (4.5; 17.6) |  | 111 | 5.4 (2; 11.4) |  | 113 | 0.9 (0; 4.8) | 112 | 0 (0; 3.2) |
| Swelling | All | 447 | 38.9 (34.4; 43.6) | 18 | 83.3 (58.6; 96.4) |  | 93 | 62.4 (51.7; 72.2) |  | 111 | 48.6 (39; 58.3) |  | 113 | 22.1 (14.9; 30.9) | 112 | 19.6 (12.7; 28.2) |
|  | Grade 3 |  |  | 18 | 22.2 (6.4; 47.6) |  | 93 | 4.3 (1.2; 10.6) |  | 111 | 4.5 (1.5; 10.2) |  | 113 | 1.8 (0.2; 6.2) | 112 | 0 (0; 3.2) |

N; number of participants with the documented dose; %; percentage of participants reporting the specified adverse event at least once; 95% CI; exact 95% confidence interval; dTpa, diphtheria-tetanus-acellular pertussis; all; all reports of the specified adverse event irrespective of intensity grade and relationship to vaccination.

For children <6 years of age, grade 3 pain was defined as crying when the limb was moved or the limb being spontaneously painful and grade 3 redness and swelling as maximum intensity of local redness/swelling with a diameter > 20 mm; for children and adults ≥ 6 years of age grade 3 pain was defined as significant pain at rest, preventing normal every day activities and redness and swelling as maximum intensity of local redness/swelling with a diameter > 50 mm.

# Supplementary figure 1. Plain language summary


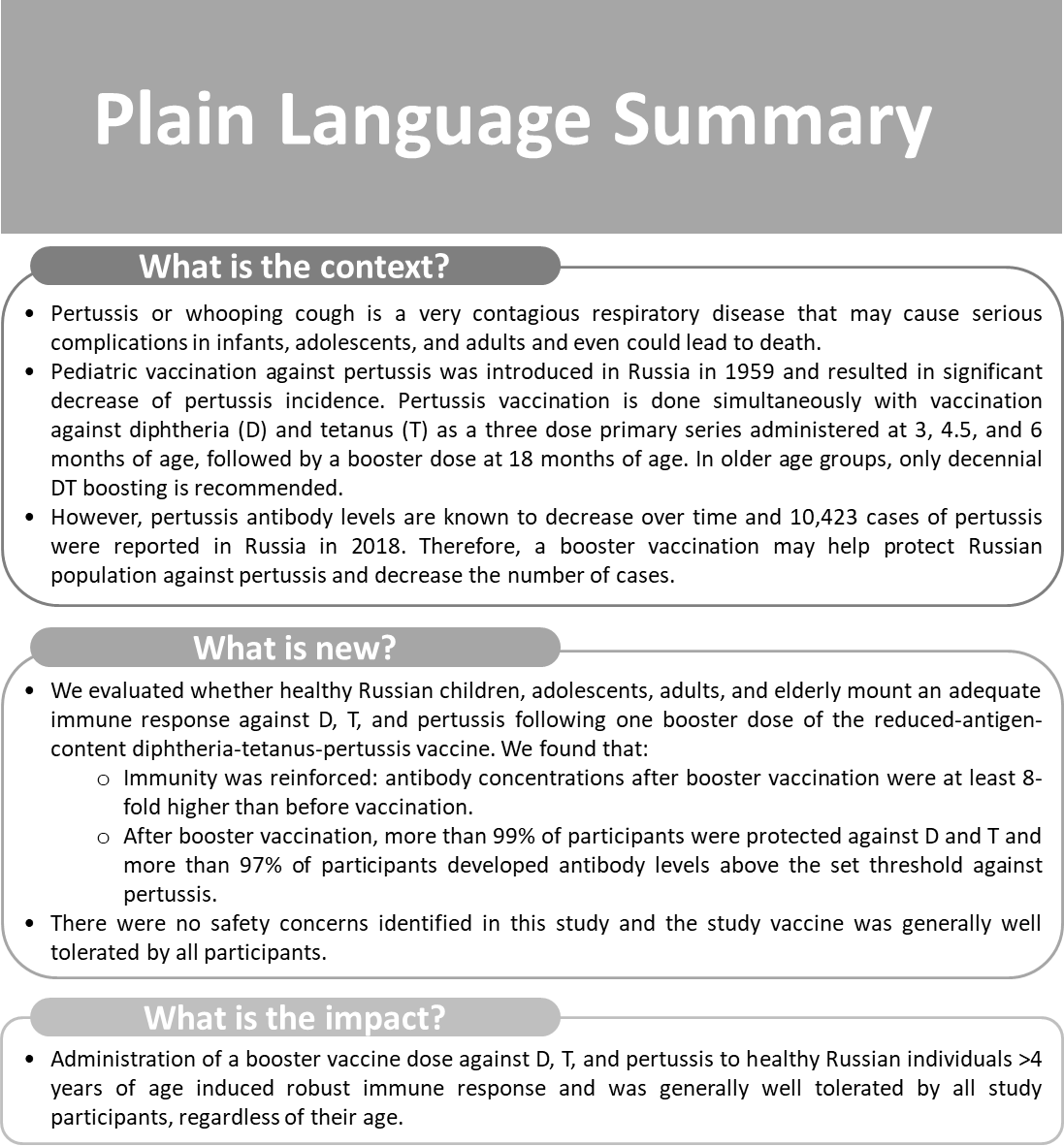


#
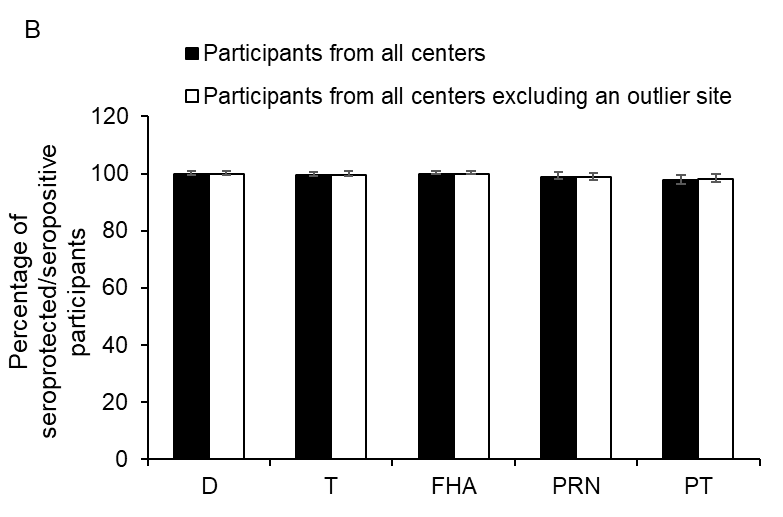

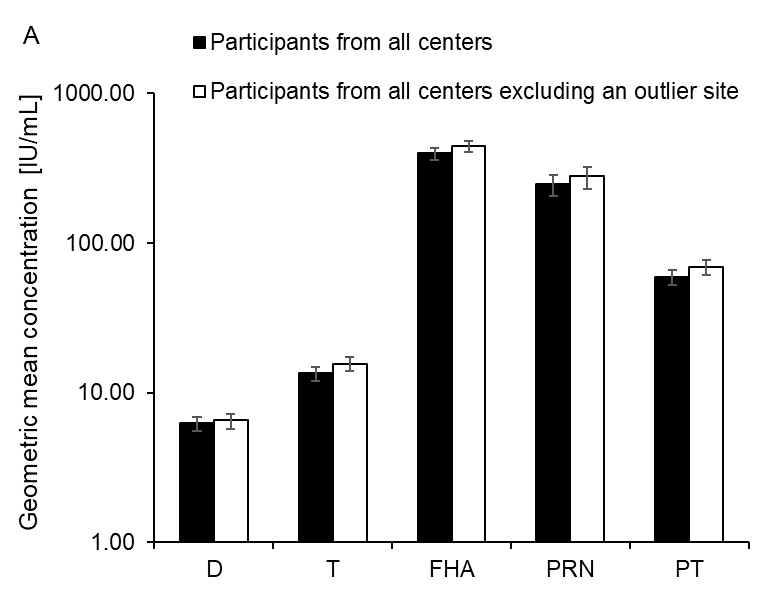
Supplementary figure 2. Geometric mean concentration (A) and seroprotection/seropositivity rate (B) one month after dTpa vaccination for anti-D, anti-T, and anti-pertussis antibodies (per protocol cohort for analysis of immunogenicity and per protocol cohort for analysis of immunogenicity excluding the outlier site)

D, diphtheria; dTpa, diphtheria-tetanus-acellular pertussis vaccine; FHA, filamentous hemagglutinin; IU, international unit; PRN, pertactin; PT, pertussis toxoid, T, tetanus. (A) Log scale in Y axis. Percentage of participants who were seroprotected (antibody concentrations equal to or above the specified seroprotection cut-off of 0.1 IU/mL for diphtheria and pertussis) /seropositive (antibody concentration equal to or above the cut-off of 2.046 IU/mL, 2.187 IU/mL and 2.693 IU/mL, for filamentous hemagglutinin, pertactin and pertussis toxoid, respectively).
